# iRECIST-based versus non-standardized free text reporting of CT scans for monitoring metastatic renal cell carcinoma: a retrospective comparison

**DOI:** 10.1007/s00432-022-03997-0

**Published:** 2022-04-14

**Authors:** Laura Schomburg, Amer Malouhi, Marc-Oliver Grimm, Maja Ingwersen, Susan Foller, Katharina Leucht, Ulf Teichgräber

**Affiliations:** 1 Department of Diagnostic and Interventional Radiology, Friedrich-Schiller-University, University Hospital Jena, Am Klinikum 1, 07747 Jena, Germany; 2 Department of Urology, Friedrich-Schiller-University, University Hospital Jena, Am Klinikum 1, 07743 Jena, Germany

**Keywords:** Immunotherapy, Disease progression, Renal cell carcinoma, Response evaluation criteria in solid tumors, Tumor burden

## Abstract

**Purpose:**

Therapy decision for patients with metastatic renal cell carcinoma (mRCC) is highly dependent on disease monitoring based on radiological reports. The purpose of the study was to compare non-standardized, common practice free text reporting (FTR) on disease response with reporting based on response evaluation criteria in solid tumors modified for immune-based therapeutics (iRECIST).

**Methods:**

Fifty patients with advanced mRCC were included in the retrospective, single-center study. CT scans had been evaluated and FTR prepared in accordance with center’s routine practice. For study purposes, reports were re-evaluated using a dedicated computer program that applied iRECIST. Patients were followed up over a period of 22.8 ± 7.9 months in intervals of 2.7 ± 1.8 months.

Weighted kappa statistics was run to assess strength of agreement. Logistic regression was used to identify predictors for different rating.

**Results:**

Agreement between FTR and iRECIST-based reporting was moderate (kappa 0.38 [95% CI 0.2–0.6] to 0.70 [95% CI 0.5–0.9]). Tumor response or progression according to FTR were not confirmed with iRECIST in 19 (38%) or 11 (22%) patients, respectively, in at least one follow-up examination. With FTR, new lesions were frequently not recognized if they were already identified in the recent prior follow-up examination (odds ratio for too favorable rating of disease response compared to iRECIST: 5.4 [95% CI 2.9–10.1].

**Conclusions:**

Moderate agreement between disease response according to FTR or iRECIST in patients with mRCC suggests the need of standardized quantitative radiological assessment in daily clinical practice.

**Supplementary Information:**

The online version contains supplementary material available at 10.1007/s00432-022-03997-0.

## Introduction

The incidence of renal cell carcinoma up to age 75 is 4.4/100 000 worldwide (2.4% of new cancer cases, 1.7% of cancer deaths) (Ferlay et al. 2015) with higher incidence rates and declining mortality in developed countries (Znaor et al. 2015). For the survival of patients, it is crucial that radiological treatment monitoring provides substantial information for therapeutic decision making. However, a recent study suggests that the value of radiologic reports considerably depends on the methodical approach of assessment (Goebel et al. 2017).

Whilst in routine clinical practice, quantitative assessment criteria for morphologic CT image interpretation are rarely standardized and the method of free text reporting (FTR) is common, in clinical studies, consideration of standardized response evaluation criteria in solid tumors (RECIST) is required. With FTR, in general, no specific criteria for evaluation are defined. Radiologists routinely refer to the most previous finding. In contrast, RECIST based reporting refers either to baseline or to nadir. Moreover, with RECIST, selection of target lesions is specified. RECIST were updated in 2009 (validated RECIST 1.1 guideline) and are mainly to be applied in case of cytotoxic chemotherapy (Eisenhauer et al. 2009) whereas immune-RECIST (iRECIST consensus guideline) were proposed in 2017 and are supposed to be applied in patients who receive immunotherapy (Seymour et al. 2017). With immunotherapy, even initial progression due to infiltration of various immune cells into the tumor with subsequent reduction or stabilization of the tumor size (pseudoprogression) is associated with prolonged survival (Ma et al. 2019; Aykan et al. 2020). Application of iRECIST for the assessment of metastatic renal cell carcinoma (mRCC) associated tumor burden, supported by semi-automated comparison using specified references (i.e.: baseline or nadir) may improve radiological assessment and reporting quality even in all day clinical practice.

The purpose of our study was to evaluate whether assessment of disease response in patients with mRCC according to common practice FTR without specified evaluation criteria sufficiently agrees with that based on software-aided application of iRECIST.

## Methods

### Study population

This retrospective, single-center study was approved by the institutional review board.

We searched the institutional medical database and included 50 consecutive patients with mRCC who had been treated with immunotherapy between January 2015 and October 2020. Patients with a measurable tumor burden at baseline according to current guidelines on tumor response criteria (RECIST 1.1 and iRECIST Eisenhauer et al. 2009; Seymour et al. 2017)), who had completed at least three follow-up examinations with contrast-enhanced CT of thorax, abdomen, and pelvis were eligible for inclusion.

### CT data acquisition

CT scans were performed using Revolution CT (GE Healthcare) with a detector width of 160 mm. After intravenous administration of a 1 ml/kg body weight bolus of contrast agent (Accupaque 350 mg, GE Healthcare) followed by a 50 ml saline flush, the imaging started with a bolus-triggered technique (monitoring frequency from 10 s after contrast injection: 1 per second; trigger threshold: an increase of 100 HU in the descending aorta; delay from trigger to initiation of scan: 15 s). For CT scans, parameters were as follows: 120 kVp, automatically set mAs values, reconstructed to a slice thickness of 3 mm and a pitch of 53.

### Image analysis

Based on morphological evaluation of CT scans, FTRs had been prepared as part of daily clinical practice by two radiologists in agreement. At least one of them had more than 5 years of experience concerning assessment of tumor burden. Overall, 58 radiologists had participated in preparation of the considered FTRs. FTRs covered both description of pathologies and clinical interpretation including indication of tumor development. Assessment criteria had not been specified. Tumor burden after a given treatment had usually been compared to the recent prior CT examination, however, reference CT was not mentioned explicitly. For study purposes, if not already done by the examiner, a resident physician of the radiology department retrospectively assigned interpretation of FTRs to one of the following four disease response categories: complete response (CR), partial response (PR), stable disease (SD), or progressive disease (PD).

In parallel, we imported the same image datasets into a commercially available semi-automatic software (mint lesion version 3.7.3, MINT Medical GmbH) (Goebel et al. 2017). A radiologist with 13 years of experience, blinded to FTRs, retrospectively selected target lesions for baseline entries. Classification of target lesions was based on specified criteria including size, number per organ system, and reproducible measurability according to RECIST 1.1 guidelines. Lesions which did not meet these criteria were classified as non-target lesions (Eisenhauer et al. 2009). Subsequently, the radiologist manually measured the longest/shortest axis of each lesion with the aid of the software. The software automatically summed up the longest diameter of non-nodal target lesions and the short axis diameter of nodal target lesions. Lesions from follow-up CT scans were detected with support of the software and measured manually. The software again calculated the sum of lesion axis diameters at every follow-up to automatically compare it with the appropriate reference and assigned the respective disease category. According to iRECIST (Seymour et al. 2017), baseline CT serves as reference to determine response to treatment (iCR or iPR) and stable disease (iSD). Nadir (smallest sum of diameters so far) serves as reference to determine progression. There are two definitions of progress: unconfirmed progressive disease (iUPD) and confirmed progressive disease (iCPD: triggered by further progress after iUPD) (Online Resource: ESM Table 1). In addition, image analysis based on RECIST 1.1 was conducted to estimate to what extent pseudoprogression contributed to different assessment of tumor development.

### Study outcome

In this study, unstandardized FTR on disease response was compared to software-supported assessment using iRECIST. The latter had been chosen as comparator because, in contrast to RECIST 1.1, iRECIST take effects of immunotherapy into account that may mimic tumor progression (pseudoprogression) (Seymour et al. 2017; Ma et al. 2019). Outcome of primary interest was strength of agreement regarding change in tumor burden according to FTR and iRECIST, quantified as weighted kappa. Secondary outcomes were proportionate agreement between the two approaches and associations of selected variables with different rating among FTR and iRECIST-based reports.

### Statistical analysis

Categorical variables are presented as counts and percentages and continuous variables as means and standard deviations. Agreement was assessed with Cohen’s kappa statistics. Amount of difference in rating was considered using weighted kappa. Strength of agreement was interpreted according to Landis and Koch (1977), (kappa ≤ 0.00: poor; 0.01–0.20: slight; 0.21–0.40: fair; 0.41–0.60: moderate; 0.61–0.80: substantial; 0.81–1.0: almost perfect agreement). Mann–Kendall test was used to determine whether agreement in tumor assessment had a trend over the series of follow-up examinations. Univariable analysis using logistic regression was applied to assess associations between selected variables with different rating of tumor burden with FTR or iRECIST. The p value threshold was adjusted for multiple testing (*p* < 0.006). Analysis was performed using StatsDirect statistical software version 2.8.0. (StatsDirect Ltd.) and XLSTAT version 2015.6.01.24026 (Addinsoft).

## Results

### Study population

A total of 50 patients (64 ± 10 years, 68.0% male sex) with mRCC were included in the study. Prognostic risk according to international metastatic renal cell carcinoma database consortium (IMDC) assessment was poor in 20% of patients. Average number of target lesions per patient was 2.4 ± 1.4. The most common sites of target and non-target lesions were lung (33.0%), lymph nodes (30.0%), kidney (7.1%), and liver (6.6%) (Table 1). Patients completed 8.4 ± 2.3 CT follow-up evaluations over a period of 22.8 ± 7.9 months. Intervals between follow-ups were 2.7 ± 1.8 months (Online Resource: ESM Table 2).

**Table 1:** Patient and disease characteristics

Patients (*n* = 50)		(%)
Age, years	64.3 ± 10.4	
Male sex	34	68
Histology		
Clear cell RCC	46	92
Non-clear cell RCC^a^	4	8
IMDC^b^ risk group		
Favorable	6	12
Intermediate	34	68
Poor	10	20
Previous immunotherapy	24	48
Previous radiotherapy	5	10
Previous nephrectomy		
Radical	41	82
Partial	4	8
R0 resection^c^		
Yes	31	69
No	9	20
Unknown	5	11
RCC size at time of diagnosis, cm	8.6 ± 3.5	
Fuhrman grade^d^ at time of diagnosis		
G1	2	4
G2	16	32
G3	19	38
G4	7	14
Unknown	6	12
T stage^e^ at time of diagnosis		
T1	10	20
T2	8	16
T3	22	44
T4	4	8
Unknown	6	12
Target lesions per patient (*n* = 119)	2.4 ± 1.4	
Non-target lesions per patient (*n* = 78)	1.6 ± 1.4	
Lesion location (*n* = 197)		
Lung	65	33.0
Lymph node	60	30.0
Kidney	14	7.1
Liver	13	6.6
Adrenal gland	10	5.1
Pancreas	10	5.1
Soft tissue	9	4.6
Pleura	8	4.1
Bones and soft tissue	4	2.0
Vena cava	2	1.0
Mamma	1	0.5
Peritoneum	1	0.5

Categorical variables are presented as counts and percentages and continuous variables as means and standard deviations

*IMDC* international metastatic renal cell carcinoma database consortium, *RCC* renal cell carcinoma

^a^Non-clear cell RCC: papillary RCC, collecting duct RCC

^b^IMDC prognostic risk was rated as follows: 0 risk factors, favorable; 1–2 risk factors, intermediate; ≥ 3 risk factors, poor (Motzer et al. 2019)

^c^No cancer cells seen microscopically at the primary tumor site

^d^Nuclear grading system based on microscopic morphology, which evaluates nuclear size, shape, and nucleolar prominence

^e^T stage describes the size and extent of the primary tumor (Lam et al. 2009)

### Free text reporting versus iRECIST-based reporting

**Fig. 1:**
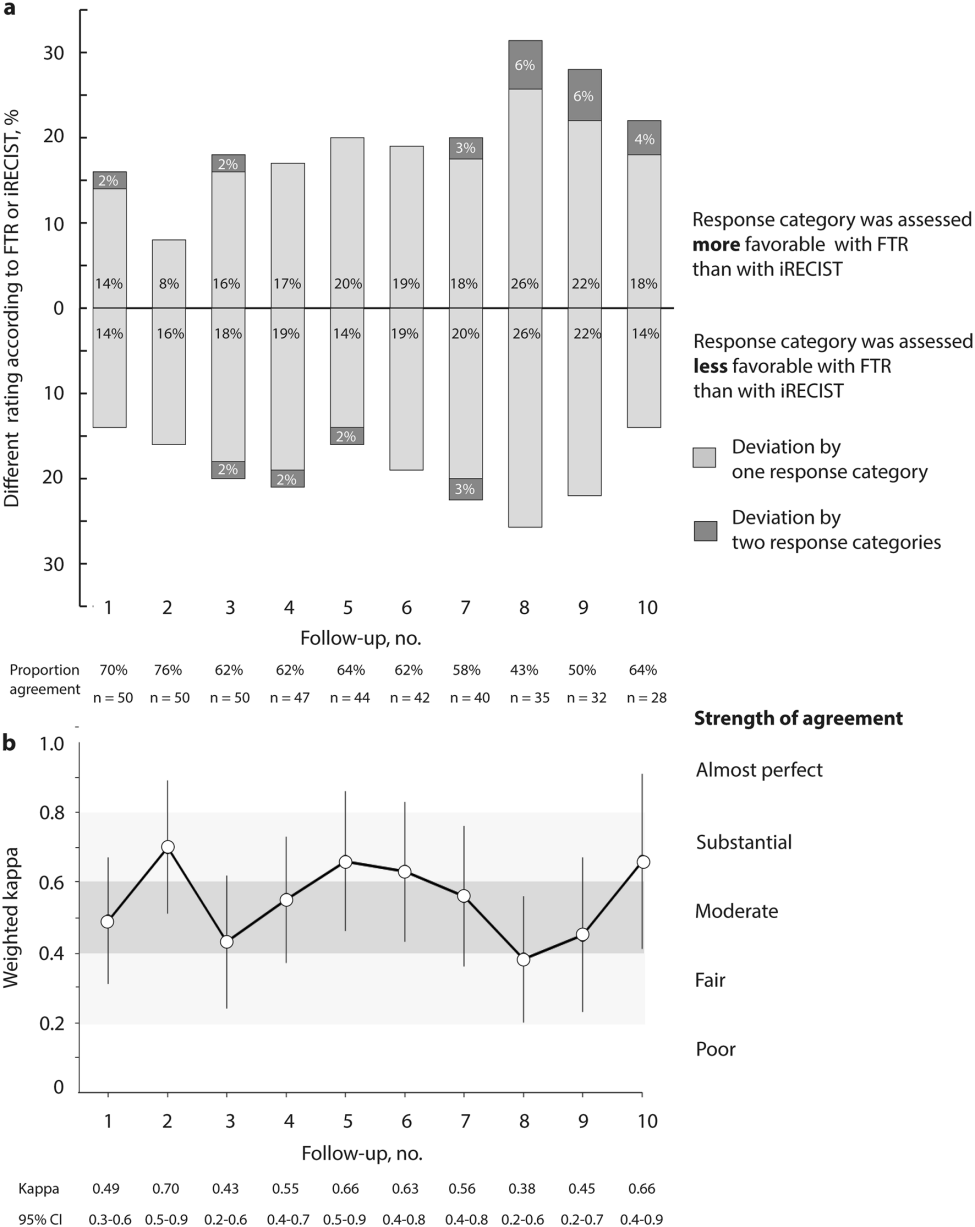
Difference in assessment of response categories between FTR and iRECIST. Proportion of different assessment (**a**) and strength of agreement (**b**) regarding response category in patients with metastatic renal cell carcinoma with either FTR or iRECIST. Strength of agreement is presented as weighted kappa with 95% confidence interval. Regarding iRECIST, unconfirmed progressive disease (iUPD) was put on the same level with confirmed progressive disease (iCPD). FTR, free text reporting; iRECIST, response evaluation criteria in solid tumors for immune-based therapeutics

Rating of change in tumor burden according to either FTR or iRECIST differed in 30–57% of patients throughout follow-ups. In 8–32% of patients, response category was assessed more favorable, and in 14–26% less favorable with FTR. Ratings differed by one (24–52% of patients) or two (0–6% of patients) categories (Fig. 1a).

Strength of agreement between FTR and iRECIST-based reports was fair to substantial throughout CT follow-up examinations. Weighted kappa ranged from 0.38 (95% CI 0.2–0.6) to 0.70 (95% CI 0.5–0.9). There was no trend in the series of weighted kappa over subsequent follow-ups (Kendall’s Tau -0.05, *p* = 0.93), (Fig. 1b). Weighted kappa increased when iRECIST was experimentally run without considering nadir or baseline but instead only referring to the respective preceding examination, analogous to the approach of FTR (0.47 [95% CI 0.29–0.65] to 0.92 [95% CI 0.71–1.12]), (Online Resource: ESM Fig. 1).

**Fig. 2:**
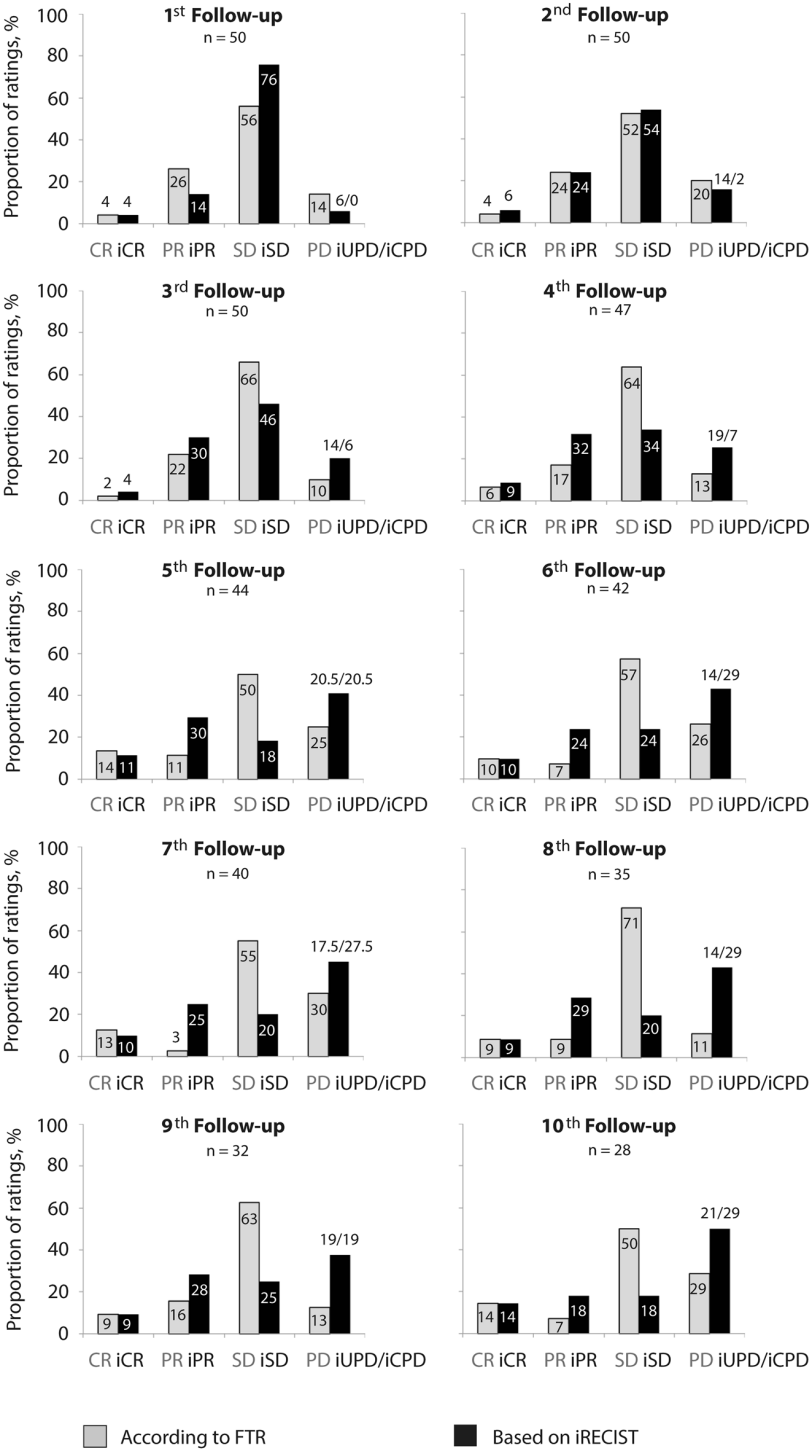
Distribution of disease response categories according to approach of reporting. CR complete response, *iCPD* immune confirmed progressive disease, *iCR* immune complete response, *iPR* immune partial response; iSD, immune stable disease, *iUPD* immune unconfirmed progressive disease, *PD* progressive disease, *PR* partial response, *SD* stable disease

With FTR, disease was preferably reported as SD at all follow-ups. Assessment of PR was 8–22 and PD was 10–32 percentage points less with FTR compared to iRECIST-based reports from the 3rd follow-up on. In contrast, with iRECIST, proportion of SD ratings declined from 76% at the 1st to 18% at the 10th follow-up. Overall, FTR on response or progression was not confirmed by iRECIST-based reports in 22% (21 of 96 CT evaluations, 19 patients) or 16% (12 of 76 CT evaluations, 11 patients), respectively (Fig. 2). Two of the patients in whom FTR indicated PD but iRECIST stated SD (2/11) underwent immediate change of treatment. However, change was due to diarrhea in one of them.

### Pseudoprogression

**Fig. 3:**
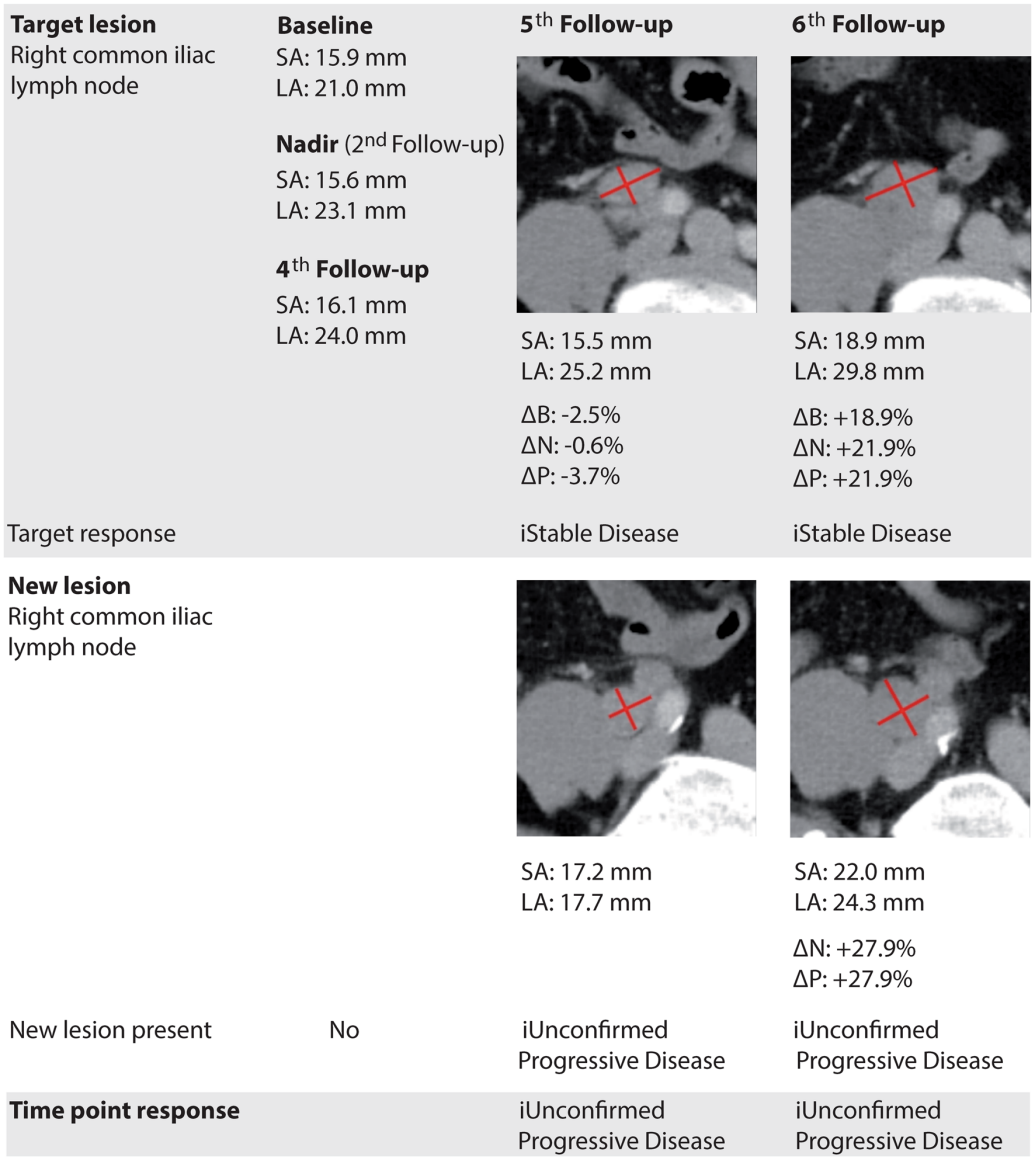
Unconfirmed progression assessed with iRECIST in a 62-year-old male patient presenting with tumorous lymph nodes. At the 6th follow-up, target lesion response was rated as stable because short axis of the lymph node increased by ≥ 20% but by < 5 mm and thus did not meet criteria for progression. New lesion assessment was rated as iUPD because measure of short axis was still > 10 mm but did not increase by ≥ 5 mm from the initial detection. Thus, progressive disease was not confirmed, and overall response status was calculated as iUPD. *B* baseline, *iUPD* immune unconfirmed progressive disease, *LA* long axis, *N* nadir, *P* previous follow-up, *SA* short axis

Agreement between assessment of change in tumor burden using RECIST 1.1 or iRECIST was almost perfect (weighted Cohen’s kappa: 0.89 (95% CI 0.63–1.14) to 1.0 (95% CI 0.81–1.19), Online Resource: ESM Fig. 2. Different assessment was caused by pseudoprogression in 5 patients (identified with iRECIST at 11 follow-ups) representing 10% of the total study cohort. Pseudoprogression was observed in lymph nodes (3 lesions), lung (3 lesions), and pleura (1 lesion). In 4 of 11 (36%) examinations, pseudoprogression was also covered by FTR. Examples for assessment of progression with iRECIST are provided in Fig. 3 and Online Resource: ESM Fig. 3.

### Predictors of different assessment

**Fig. 4:**
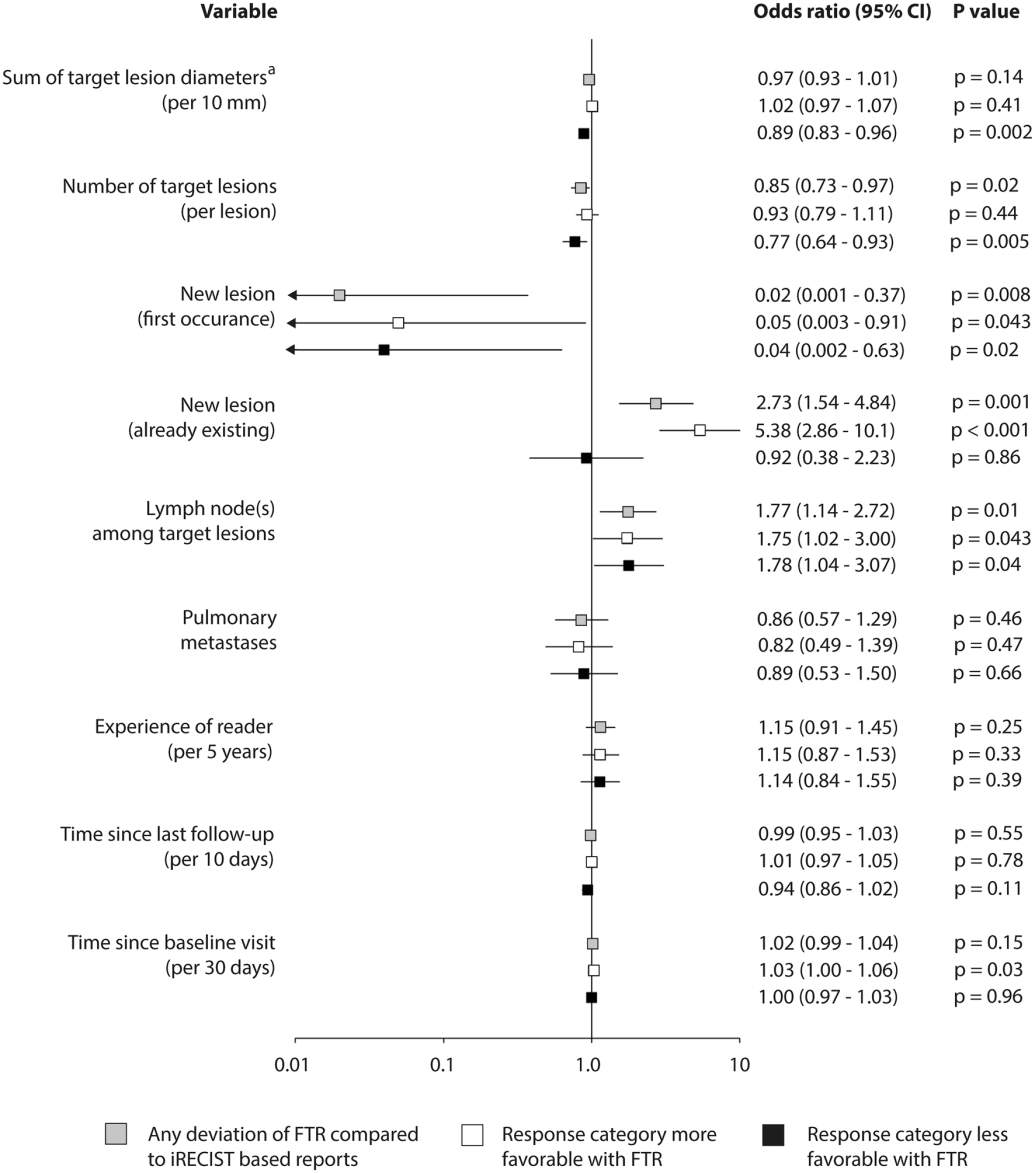
Association of selected variables with assessment of disease response category depending on the approach of reporting. Based on univariable logistic regression ^a^For lymph nodes, actual short axis measurement was included in the sum diameter

Univariable analysis revealed that first occurrence of new lesions decreased the odds of different assessment according to FTR compared to iRECIST (odds ratio [OR] 0.02 (95% CI 0.001–0.4). In contrast, the presence of already existing new lesions raised the odds that disease response was assessed as better according to FTR than to iRECIST by more than fivefold (OR 5.4 (95% CI 2.9–10.1). Both an increase in the sum of target lesion diameters and in the number of target lesions decreased the odds of disease response of being assessed as lower according to FTR compared to iRECIST (OR 0.9 per 10 mm [95% CI: 0.8–1.0] and OR 0.8 per lesion [95% CI 0.6–0.9], respectively). Involvement of lymph nodes nearly doubled the odds of different assessment (OR 1.77 (95% CI 1.1–2.7). Every month from baseline increased the odds that disease response was assessed as better according to FTR than to iRECIST (OR 1.0 per 30 days from baseline [95% CI 1.0–1.1]) (Fig. 4).

## Discussion

We conducted a retrospective comparison of FTR with reporting based on software-supported application of iRECIST to assess change in tumor burden on grounds of anatomically unidimensional CT scan measurement in patients with mRCC. Agreement between both approaches was only moderate. According to unstandardized FTR, new lesions which were already present in recent prior follow-ups were frequently not recognized as such. Different assessment following FTR compared to iRECIST was more frequently seen if lymph nodes were target lesions.

In daily clinical practice, evaluation of tumor burden in addition to symptomatic criteria represents a crucial parameter for therapy control. Although, the iRECIST guideline basically does not apply for clinical decision making (Seymour et al. 2017) and criteria still need to be validated, standardized response criteria could facilitate objectivity of assessment even in daily practice.

A previous trial on tumors of various origins (Goebel et al. 2017) reported on fair to moderate agreement between FTR and RECIST 1.1 based reports. In most cases, different reporting with FTR was attributed to assignment of even minor changes in tumor burden to PR or PD instead of SD. Another reason for discrepancies were comparisons to the most recent prior follow-up examinations instead to baseline or nadir. The latter was confirmed in our study. In addition, with FTR, new lesions that were already present were frequently not recognized as still existing new lesions, with the result of a too favorable rating. In case of lymph nodes, discrepancies might be based on lesion selection (iRECIST: a maximum of two nodes in total, even from different nodal basins) and measurement (iRECIST: at least 15 mm in short axis). Additionally, we found that the odds of too worse rating with FTR was reduced with increasing number and sum of diameters of target lesions. However, it seems conclusive that in advanced disease, worse rating is more frequently appropriate.

Assessment of pseudoprogression with iRECIST can only be determined with certainty after 4 weeks from the first detection of iUPD. After 4–8 weeks, disease could further progress and trigger iCPD or could stay progredient compared to nadir (iUPD) or show stable disease (iSD), partial response (iPR), or complete response (iCR) compared to baseline. This new assessment should prevent patients to be withdrawn from immunotherapy in case of pseudoprogression. An earlier study found that continued immunotherapy beyond iUPD can prolong survival (George et al. 2016). Such a review of assessment is not automatically provided with FTR, however, could be visualized using longitudinal analysis and graphical methods (Shen et al. 2014). In our study, FTR investigators identified pseudoprogression in some of the patients with increased lymph node axis despite of decreased sum of parenchymal lesion diameters. Frequency of pseudoprogression, determined with iRECIST was similar to previous findings in patients with mRCC (9–14%) (Queirolo et al. 2017).

For unexperienced readers, even RECIST contains pitfalls that may origin in baseline lesion selection, reassessment of lesions, and identification of new lesions (Abramson et al. 2015; Keil et al. 2014) identified the choice of target lesions as major source of disagreement between readers, even with consideration of RECIST 1.1. Thus, target lesion selection probably remains a subjective confounder in the assessment of tumor development. Target lesions should be unequivocally metastases, but not postoperative seroma or granulation tissue. Lesions within a radiation field should not be selected as target lesions unless progression is demonstrated. At follow-up, lesions should be remeasured in the same phase of contrast. Finally, even in case of axis shift, it is required to remeasure the true long (parenchymal lesions) or short axis (nodal lesions), respectively (Abramson et al. 2015).

RECIST/iRECIST are well described and known in clinical practice, however, rarely implemented. This may be due to need for assignment of the appropriate reference CT which requires request for baseline information including treatment modalities from oncologists. Moreover, reproducibility is supposed to increase when reference and follow-up CT scan is read by the same radiologist (Muenzel et al. 2012; Olthof et al. 2018). According to Krajewski et al. intra-rater variability in CT tumor size measurement ensures reproducibility of 10% tumor shrinkage measured by a single radiologist (Krajewski et al. 2014). However, this approach might be difficult to apply in small radiology departments. Thus, software-aided application of RECIST/iRECIST, however, can be a sufficient and time-saving support to increase reproducibility of the reporting. From this one might conclude that the large number of radiologists who prepared FTRs might have contributed to the decreased agreement with iRECIST-based reports conducted by a single radiologist.

This study has some limitations. First, assessment criteria applied for FTR were not reported. In addition, assignment of FTR interpretation to disease categories for study purposes was not conducted by oncologists, the actual receivers, but by radiologists. Anyway, assignment left scope for interpretation. Furthermore, only a single reader conducted assessment according to iRECIST and we did neither systematically consider treatment decisions nor clinical outcomes. However, as observed in patients in whom FTR indicated progressive- but iRECIST found stable disease, treatment decision, was not necessarily associated with CT assessment. Finally, our small-scale trial was conducted retrospectively at a single center and thus, should be considered as exploratory.

## Conclusions

In conclusion, utility of radiological assessment for treatment stratification increases with objectivity and traceability of reports. Standardized assessment criteria constitute a sound basis for quantitative morphologic evaluation of disease development in patients with mRCC. Additional text-only qualitative reports may be better suited to be seen by the patient (Travis et al. 2014). Due to unsatisfactory agreement between FTR and iRECIST-based reporting, we recommend extending application of iRECIST from clinical studies to routine clinical practice. Dedicated software may support implementation.

## Supplementary Information

Below is the link to the electronic supplementary material.

Supplementary file1 (DOCX 5899 KB)

## Data Availability

The datasets analysed during the current study are not publicly available for protection purposes but are available from the corresponding author on reasonable request.

## References

[CR1] Abramson RG, Mcghee CR, Lakomkin N, Arteaga CL (2015). Pitfalls in RECIST data extraction for clinical trials: beyond the basics. Acad Radiol.

[CR2] Aykan NF, Ozatli T (2020). Objective response rate assessment in oncology: current situation and future expectations. World J Clin Oncol.

[CR3] Eisenhauer EA, Therasse P, Bogaerts J, Schwartz LH, Sargent D, Ford R, Dancey J, Arbuck S, Gwyther S, Mooney M, Rubinstein L, Shankar L, Dodd L, Kaplan R, Lacombe D, Verweij J (2009). New response evaluation criteria in solid tumours: revised RECIST guideline (version 1.1). Eur J Cancer.

[CR4] Ferlay J, Soerjomataram I, Dikshit R, Eser S, Mathers C, Rebelo M, Parkin DM, Forman D, Bray F (2015). Cancer incidence and mortality worldwide: sources, methods and major patterns in GLOBOCAN 2012. Int J Cancer.

[CR5] George S, Motzer RJ, Hammers HJ, Redman BG, Kuzel TM, Tykodi SS, Plimack ER, Jiang J, Waxman IM, Rini BI (2016). Safety and efficacy of nivolumab in patients with metastatic renal cell carcinoma treated beyond progression: a subgroup analysis of a randomized clinical trial. JAMA Oncol.

[CR6] Goebel J, Hoischen J, Gramsch C, Schemuth HP, Hoffmann AC, Umutlu L, Nassenstein K (2017). Tumor response assessment: comparison between unstructured free text reporting in routine clinical workflow and computer-aided evaluation based on RECIST 1.1 criteria. J Cancer Res Clin Oncol.

[CR7] Keil S, Barabasch A, Dirrichs T, Bruners P, Hansen NL, Bieling HB, Brummendorf TH, Kuhl CK (2014). Target lesion selection: an important factor causing variability of response classification in the response evaluation criteria for solid tumors 1.1. Invest Radiol.

[CR8] Krajewski KM, Nishino M, Franchetti Y, Ramaiya NH, Van Den Abbeele AD, Choueiri TK (2014). Intraobserver and interobserver variability in computed tomography size and attenuation measurements in patients with renal cell carcinoma receiving antiangiogenic therapy: implications for alternative response criteria. Cancer.

[CR9] Lam JS, Klatte T, Breda A (2009). Staging of renal cell carcinoma: current concepts. Indian J Urol.

[CR10] Landis JR, Koch GG (1977). The measurement of observer agreement for categorical data. Biometrics.

[CR11] Ma Y, Wang Q, Dong Q, Zhan L, Zhang J (2019). How to differentiate pseudoprogression from true progression in cancer patients treated with immunotherapy. Am J Cancer Res.

[CR12] Motzer RJ, Jonasch E, Michaelson MD, Nandagopal L, Gore JL, George S, Alva A, Haas N, Harrison MR, Plimack ER, Sosman J, Agarwal N, Bhayani S, Choueiri TK, Costello BA, Derweesh IH, Gallagher TH, Hancock SL, Kyriakopoulos C, Lagrange C, Lam ET, Lau C, Lewis B, Manley B, Mccreery B, Mcdonald A, Mortazavi A, Pierorazio PM, Ponsky L, Redman BG, Somer B, Wile G, Dwyer MA, Cgc HLJ, Zuccarino-Catania G (2019). NCCN guidelines insights: kidney cancer, version 2.2020. J Natl Compr Canc Netw.

[CR13] Muenzel D, Engels HP, Bruegel M, Kehl V, Rummeny EJ, Metz S (2012). Intra- and inter-observer variability in measurement of target lesions: implication on response evaluation according to RECIST 1.1. Radiol Oncol.

[CR14] Olthof AW, Borstlap J, Roeloffzen WW, Callenbach PMC, Van Ooijen PMA (2018). Improvement of radiology reporting in a clinical cancer network: impact of an optimised multidisciplinary workflow. Eur Radiol.

[CR15] Queirolo P, Spagnolo F (2017). Atypical responses in patients with advanced melanoma, lung cancer, renal-cell carcinoma and other solid tumors treated with anti-PD-1 drugs: a systematic review. Cancer Treat Rev.

[CR16] Seymour L, Bogaerts J, Perrone A, Ford R, Schwartz LH, Mandrekar S, Lin NU, Litiere S (2017). iRECIST: guidelines for response criteria for use in trials testing immunotherapeutics. Lancet Oncol.

[CR17] Shen Y, Anderson A, Sinha R, Li Y (2014). Joint modeling tumor burden and time to event data in oncology trials. Pharm Stat.

[CR18] Travis AR, Sevenster M, Ganesh R, Peters JF, Chang PJ (2014). Preferences for structured reporting of measurement data: an institutional survey of medical oncologists, oncology registrars, and radiologists. Acad Radiol.

[CR19] Znaor A, Lortet-Tieulent J, Laversanne M, Jemal A, Bray F (2015). International variations and trends in renal cell carcinoma incidence and mortality. Eur Urol.

