# Supplementary material for: iRECIST-based versus non-standardized free text reporting of CT scans for monitoring metastatic renal cell carcinoma: a retrospective comparison
Source: J Cancer Res Clin Oncol. 2022 Apr 14;148(8):2003–12. doi: 10.1007/s00432-022-03997-0 (PMC9294024; doi:10.1007/s00432-022-03997-0)
Supplement: Supplementary file 1 — Supplementary file1 (DOCX 5899 KB) [file 432_2022_3997_MOESM1_ESM.docx]

**Supplemental material**

**Journal of Cancer Research and Clinical Oncology**

**iRECIST-based versus non-standardized free text reporting of CT scans for monitoring metastatic renal cell carcinoma: a retrospective comparison**

**Laura Schomburg^1^, Amer Malouhi^1^, Marc-Oliver Grimm^1^, Maja Ingwersen^1^, Susan Foller^2^, Katharina Leucht^2^, Ulf Teichgräber^1^**

^1^Department of Diagnostic and Interventional Radiology, Friedrich-Schiller-University, University Hospital Jena, Jena, Germany

^2^Department of Urology, University Hospital Jena, Jena, Germany

**Corresponding author**

Prof. Dr. Ulf Teichgräber, MD

Friedrich-Schiller-University

Jena University Hospital

Department of Diagnostic and Interventional Radiology

Am Klinikum 1, 07747 Jena, Germany

Telephone: +49 3641 9324832

E-mail: [ulf.teichgraeber@med.uni-jena.de](mailto:ulf.teichgraeber@med.uni-jena.de)

| **ESM Table 1** Time point response according to iRECIST (Seymour et al. 2017) | | | | | |
| --- | --- | --- | --- | --- | --- |
| Target lesion | Non-Target lesion | New lesion | | Time point response according to iRECIST | |
|  |  |  | No prior iUPD | | Prior iUPD |
| iCR | iCR | No | iCR | | iCR |
| iCR | Non-iCR/  Non-iUPD | No | iPR | | iPR |
| iPR | Non-iCR/  Non-iUPD | No | iPR | | iPR |
| iSD | Non-iCR/  Non-iUPD | No | iSD | | iSD |
| iUPD with no change or decrease from last time point | iUPD with no change or decrease from last time point | Yes | NA | | New lesions confirm iCPD if they were previously identified and increase in size (≥ 5 mm in SOM) or number. If no change in new lesions from last time point, it remains iUPD |
| iSD/iPR/iCR | iUPD | No | iUPD | | Remains iUPD unless iCPD confirmed based on further increase in size of non-target lesion |
| iUPD | Non-iCR/ Non-iUPD/  iCR | No | iUPD | | Remains iUPD unless iCPD confirmed based on further increase of target lesion in SOM of at least 5 mm |
| iUPD | iUPD | No | iUPD | | Remains iUPD unless iCPD confirmed based on further increase in previously identified target lesion (≥ 5 mm in SOM) and / or non-target lesion iUPD |
| iUPD | iUPD | Yes | iUPD | | Remains iUPD unless iCPD confirmed based on further increase in previously identified target lesion (≥ 5 mm in SOM) and / or previously identified non-target lesion iUPD and /or size or number of new lesions previously identified |
| Non-iUPD/PD | Non-iUPD/PD | Yes | iUPD | | Remains iUPD unless iCPD confirmed based on increase in size or number of new lesions previously identified |

iCPD: immune-related confirmed progressive disease; iCR: immune-related complete response; iPR: immune-related partial response; iRECIST: immune-related response evaluation criteria in solid tumors; iSD: immune-related stable disease; iUPD: immune-related unconfirmed progressive disease; SOM: sum of measures

| **ESM Table 2** Follow-up examinations | | |
| --- | --- | --- |
| Follow-up | Patients completed follow-up | Time from preceding examination, months |
| 1^st^ | 50 (100%) | 2.8 ± 1.7 |
| 2^nd^ | 50 (100%) | 2.6 ± 1.1 |
| 3^rd^ | 50 (100%) | 2.7 ± 3.5 |
| 4^th^ | 47 (94%) | 2.4 ± 0.9 |
| 5^th^ | 44 (88%) | 2.5 ± 0.9 |
| 6^th^ | 42 (84%) | 3.0 ± 1.5 |
| 7^th^ | 40 (80% | 2.7 ± 1.0 |
| 8^th^ | 35 (70%) | 2.7 ± 1.0 |
| 9^th^ | 32 (64%) | 3.0 ± 1.5 |
| 10^th^ | 28 (56%) | 3.0 ± 1.5 |


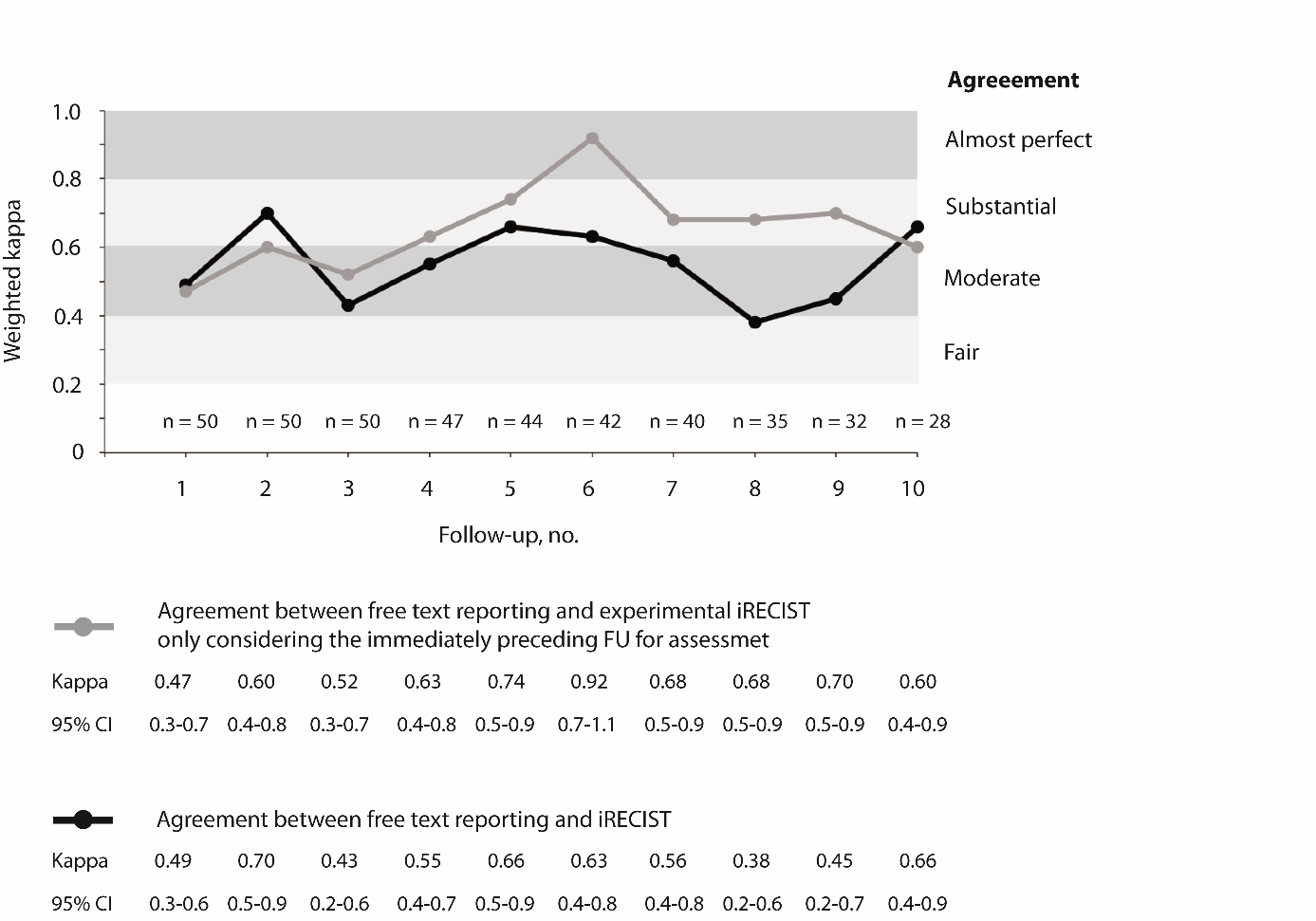


**ESM Fig. 1** Agreement on tumor response using either iRECIST or experimental iRECIST only considering the preceding follow-up. Strength of agreement is presented as weighted kappa with 95% confidence interval. iRECIST: immune-related response evaluation criteria in solid tumors.


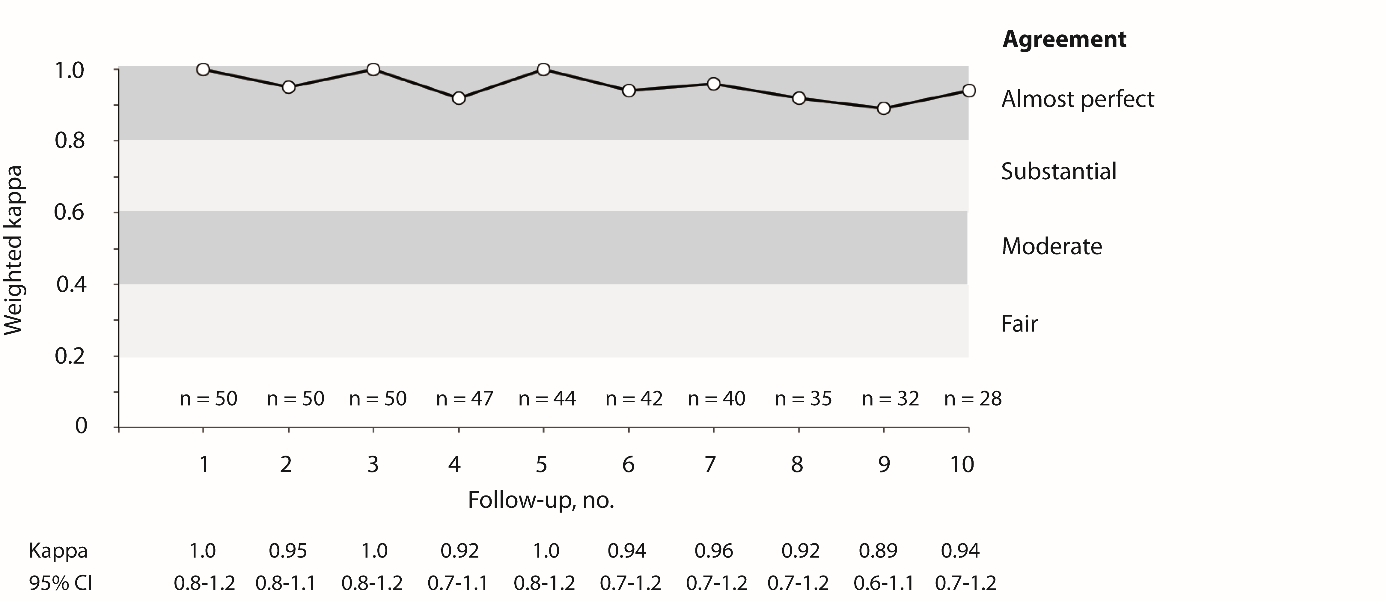


**ESM Fig. 2** Strength of agreement in tumor response between assessment using RECIST 1.1 or iRECIST. Strength of agreement is presented as weighted kappa with 95% confidence interval. iRECIST: immune-related response evaluation criteria in solid tumors.

**
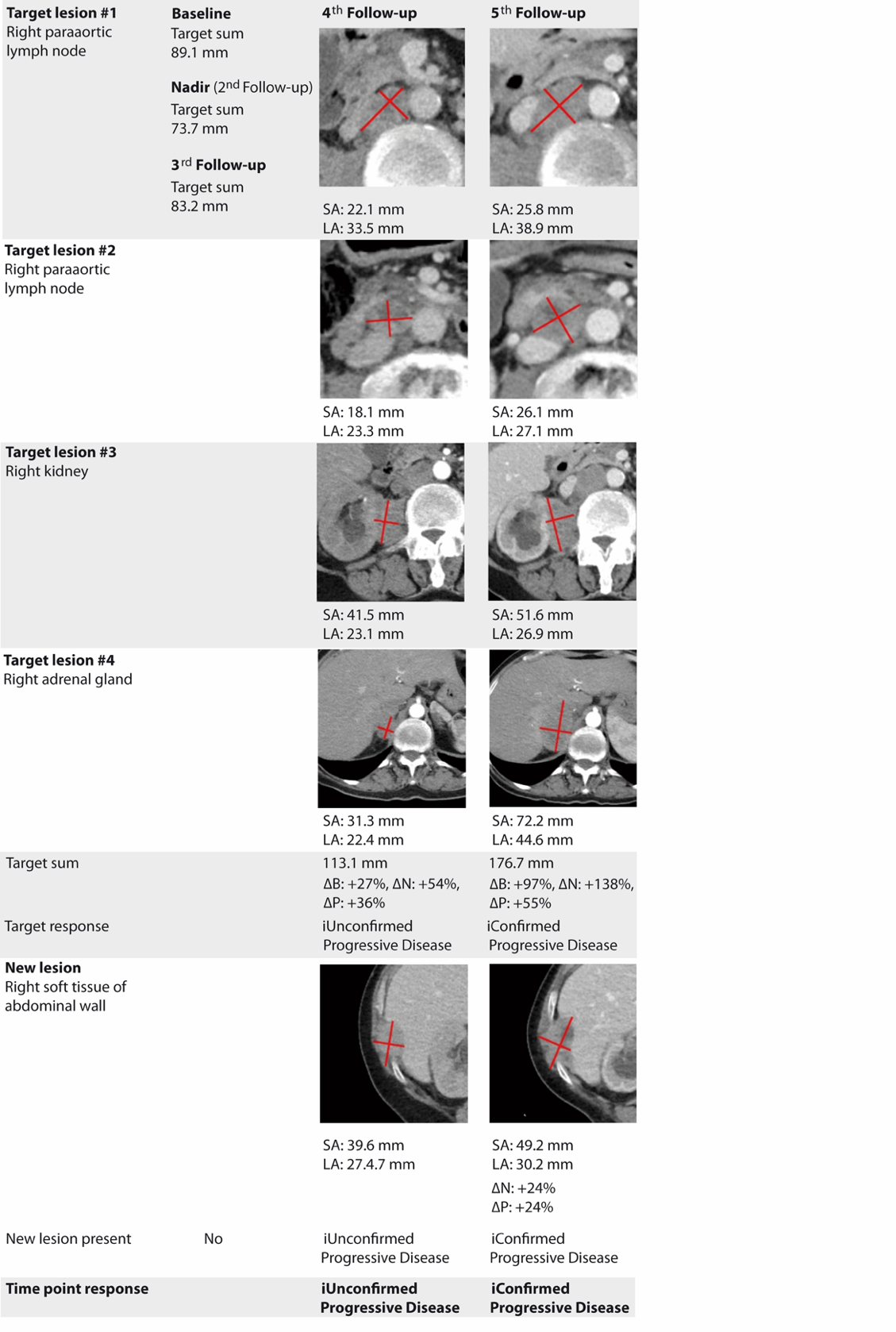
**

**ESM Fig. 3** Progressive disease assessed with iRECIST in a 57-year-old female patient presenting with renal cell carcinoma. Progressive disease was confirmed at the 5^th^ follow-up because a further increase of at least 5 mm from iUPD at the 4^th^ follow-up in both target sum diameter and new lesion diameter. B, baseline; iUPD: immune-related unconfirmed progressive disease; LA: long axis; N: nadir; P: previous follow-up; SA: short axis.

**Supplemental References**

Seymour L, Bogaerts J, Perrone A, Ford R, Schwartz LH, Mandrekar S, Lin NU & Litiere S (2017) iRECIST: guidelines for response criteria for use in trials testing immunotherapeutics. Lancet Oncol 18:e143-e152.
